# Sympathetic autonomic dysfunction and impaired cardiovascular performance in higher risk surgical patients: implications for perioperative sympatholysis

**DOI:** 10.1136/openhrt-2015-000268

**Published:** 2015-10-19

**Authors:** John Whittle, Alexander Nelson, James M Otto, Robert C M Stephens, Daniel S Martin, J Robert Sneyd, Richard Struthers, Gary Minto, Gareth L Ackland

**Affiliations:** 1 Division of Medicine, Department of Clinical Physiology, University College London, London, UK; 2 UCL Medical School, University College London, London, UK; 3 Division of Surgery and Interventional Science, University College London, Royal Free Hospital, London, UK; 4 Department of Anaesthesia, University College London Hospitals NHS Trust, London, UK; 5 Plymouth University, Peninsula Schools of Medicine and Dentistry, Plymouth, London, UK; 6 Department of Neuroscience, Physiology and Pharmacology, Centre for Cardiovascular and Metabolic Neuroscience, University College London, London, UK; 7 William Harvey Research Institute, Queen Mary University of London, London, UK

**Keywords:** MYOCARDIAL ISCHAEMIA AND INFARCTION (IHD)

## Abstract

**Objective:**

Recent perioperative trials have highlighted the urgent need for a better understanding of why sympatholytic drugs intended to reduce myocardial injury are paradoxically associated with harm (stroke, myocardial infarction). We hypothesised that following a standardised autonomic challenge, a subset of patients may demonstrate excessive sympathetic activation which is associated with exercise-induced ischaemia and impaired cardiac output.

**Methods:**

Heart rate rise during unloaded pedalling (zero workload) prior to the onset of cardiopulmonary exercise testing (CPET) was measured in 2 observation cohorts of elective surgical patients. The primary outcome was exercise-evoked, ECG-defined ischaemia (>1 mm depression; lead II) associated with an exaggerated increase in heart rate (EHRR ≥12 bpm based on prognostic data for all-cause cardiac death in preceding epidemiological studies). Secondary outcomes included cardiopulmonary performance (oxygen pulse (surrogate for left ventricular stroke volume), peak oxygen consumption (VO_2peak_), anaerobic threshold (AT)) and perioperative heart rate.

**Results:**

EHRR was present in 40.4–42.7% in both centres (n=232, n=586 patients). Patients with EHRR had higher heart rates perioperatively (p<0.05). Significant ST segment depression during CPET was more common in EHRR patients (relative risk 1.7 (95% CI 1.3 to 2.1); p<0.001). EHRR was associated with 11% (95%CI 7% to 15%) lower predicted oxygen pulse (p<0.0001), consistent with impaired left ventricular function.

**Conclusions:**

EHRR is common and associated with ECG-defined ischaemia and impaired cardiac performance. Perioperative sympatholysis may further detrimentally affect cardiac output in patients with this phenotype.

**Key questions**

**What is already known about this subject?**

- POISE-1 and POISE-2 trials reported that sympatholytic drugs (metoprolol, clonidine) aimed at reducing perioperative myocardial infarction paradoxically increase the risk of hypotension and, for metoprolol (POISE-1), death. Patients with propensity for tachycardia are more likely to receive such drugs, both in trials and routine perioperative practice.

**What does this study add?**

- Using cardiopulmonary exercise testing, we identify patients with a propensity for tachycardia in whom cardiac performance is already significantly impaired. Administering sympatholytic drugs to these patients would be expected to further compromise cardiac output, resulting in hypotension and consequently further deleterious outcomes.

**How might this impact on clinical practice?**

- Identifying patients with this exaggerated tachycardia phenotype will enable more personalised perioperative monitoring and treatment in an effort to gain the benefit of sympatholysis (reduced myocardial injury) while mitigating risks (hypotension).

## Introduction

Perioperative pharmacological interventions aimed at attenuating sympathetic activation to reduce myocardial ischaemia^1^ have met with apparently paradoxical results. Most notably, the largest series of randomised clinical trials—Perioperative Ischemic Evaluation (POISE)-1^2^ and POISE-2^3^—found that both metoprolol and clonidine resulted in more frequent episodes of hypotension. Sympatholysis-induced haemodynamic instability may result in reduced cardiac output, suboptimal organ perfusion, and consequently may explain the increase in stroke^2^ and non-fatal cardiac arrest.^3^ Thus, the trade-off between the therapeutic benefit and detrimental off-target effects associated with perioperative sympatholysis requires further investigation.^4–6^ These data also suggest that identifying patients at risk of more extreme, or persistent, sympathetic activation could improve the risk-benefit ratio of perioperative sympatholysis through a more targeted approach.^7^

As a potent trigger of increased heart rate and acute endothelial dysfunction,^8^ exaggerated sympathetic outflow following minor stress may be an important—though underappreciated—contributor to postoperative morbidity. Excessive sympathoadrenal activation directly causes catecholaminergic-mediated impairment of cardiac, regulatory mechanisms that contribute to the pathophysiology of diverse disease states.^9–14^ A minority of apparently otherwise healthy individuals who exhibit increases in heart rate as a result of the stress evoked by the thought of vigorous exercise are at increased risk of sudden cardiac, and all-cause, death.^15^

We therefore hypothesised that exaggerated heart rate increases prior to the onset of routinely performed preoperative cardiopulmonary exercise testing (CPET) would be associated with ECG evidence for ischaemia, impaired cardiopulmonary performance and inferior postoperative outcome.

## Methods

### Patient populations

Patients were enrolled at University College London Hospitals and Derriford Hospital, Plymouth, UK, having obtained IRB approval (MREC: 11/H0805/58). Informed written consent was obtained from patients undergoing preoperative CPET as routinely requested by their clinical teams prior to major elective surgery. Adherence to STROBE guidelines is documented in online supplementary table S1. Inclusion criteria were any surgical patient referred for CPET by their primary surgical and/or anaesthesia team. Exclusion criteria were according to American Thoracic Society (ATS) guidelines.^16^

### Cardiopulmonary exercise testing

**Figure 1:**
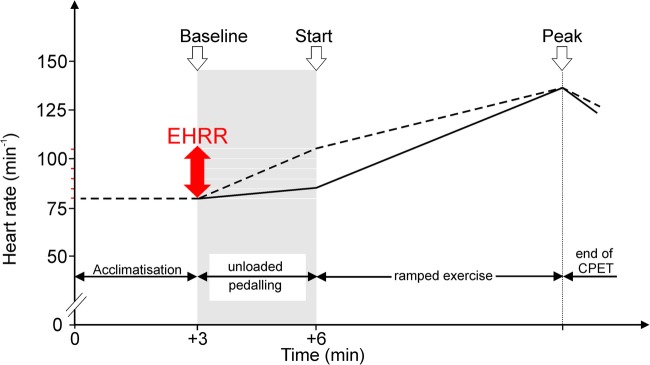
Schematic showing different phases of CPET and variables recorded. CPET, cardiopulmonary exercise testing; EHRR, exaggerated heart rate rise.

Patients completed symptom-limited maximal CPET as part of their routine preoperative assessment on a stationary cycle ergometer (Zan, nSpire, Colorado, USA; Lode, Groningen, the Netherlands). Heart rate readings were obtained via ECG with the patient sitting on the cycle ergometer. Figure 1 summarises the different stages of the CPET protocol. Patients acclimatised by sitting on the cycle ergometer for 3 min, prior to the start of exercise. Patients then undertook 3 min of unloaded pedalling, prior to the initiation of ramped exercise. Non-invasive blood pressure was measured at the start (zero workload) and at the end of CPET. We assessed heart rate rise as the difference between the heart rate at rest and the heart rate measured just before starting loaded pedalling during the exercise test protocol (ie, after 3 min of unloaded (0 W) exercise). We analysed these data by quartiles, and also defined EHRR as an abnormal exaggerated heart rate ≥12 bpm,^15^ based on previous data showing an association between stress-evoked increases in heart rate before the onset of exercise and an increased risk of sudden cardiac and all-cause cardiovascular death.^15^ All EHRR data were analysed blinded to outcomes.

### Assessment of exercise-evoked ischaemia

Continuous 12-lead ECG recordings were made throughout the CPET period to enable the detection of ischaemia and/or development of dysrhythmias. ST-segment depression was quantified in lead II, which is superior for detection of atrial dysrhythmias and more easily obtained with conventional monitors.^17^ Lead II ST changes were defined as abnormal when ST depression of 0.1 mV (1 mm) or more occurred, in accordance with current American College of Cardiology guidelines^18^ and consistent with previous studies identifying that ST-segment depression to levels ≥1 mm independently predict future cardiac events in asymptomatic populations.^19^ We also assessed ST changes by heart rate adjustment, which increases the diagnostic accuracy of the exercise ECG.^20^ The ST–heart rate (ST/HR) index was therefore calculated, by dividing the difference in ST depression at peak exercise by the exercise-induced increase in heart rate. The development at any time during the CPET of atrial and/or ventricular dysrhythmias, including ectopic beats, was also noted.

### CPET performance

Anaerobic threshold (AT), which is associated with increased postoperative morbidity and mortality,^21–26^ was assessed. AT was determined by two independent assessors blinded to EHRR and according to published guidelines using the modified V-slope method and confirmed by ventilatory equivalents for carbon dioxide 
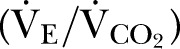
 and oxygen 
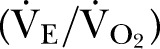
.^27^ ^28^ Peak oxygen consumption 
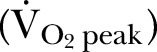
, oxygen pulse and 
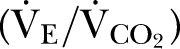
 were also recorded. Age, gender and weight-specific predicted values were calculated for 
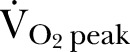
29 and oxygen pulse,^30^ a robust measure of left ventricular stroke volume.^31^ ^32^

### Perioperative heart rate

We interrogated serial heart rates in patients (n=54) who had heart rate rise measured during CPET as part of their enrolment into the Post-Operative Morbidity-Oxygen delivery trial (POM-O) randomised controlled trial.^33^ Mean heart rate over a 5 min period was compared preoperatively (5 min prior to induction), intraoperatively (end of operation prior to cessation of anaesthesia) and recovery (∼30 min after extubation, prior to start of trial protocol).

### Statistics

Baseline characteristics of participants were compared according to quartile using analysis of variance (ANOVA; quantitative variables). For continuous data, tests for skewness were performed to assess normality and, where appropriate, the data were analysed with ANOVA. Non-parametric data were analysed with the Kruskal-Wallis test. The Gehan-Breslow-Wilcoxon method was used to analyse hospital stay since this gives more weight to accelerated hospital discharge at earlier time points, which is most relevant to the hypothesis that sympathetic autonomic dysfunction increases the risk of early postoperative morbidity and hence delayed discharge. All reported p values are two-sided, with significance set at p≤0.05. Statistical analyses were performed using NCSS V.8 (Kaysville, Utah, USA).

### Sample size calculation

The primary outcome was ST depression ≥1 mm detected during CPET. Using the VISION study definition of myocardial injury after non-cardiac surgery as a guide,^1^ which reported a myocardial infarction rate of ∼8% patients undergoing non-cardiac surgery, we estimated that significant ST depression would occur in twice as many patients with EHRR. Having established a prevalence of EHRR ∼34% in the Plymouth cohort before analysing ST changes, we catered for a 10% drop-out rate (failure to complete CPET, difficulty in determining AT and poor quality ECG data) by aiming to recruit 895 patients undergoing CPET (α of 0.05; power of 80%).

## Results

**Figure 2:**
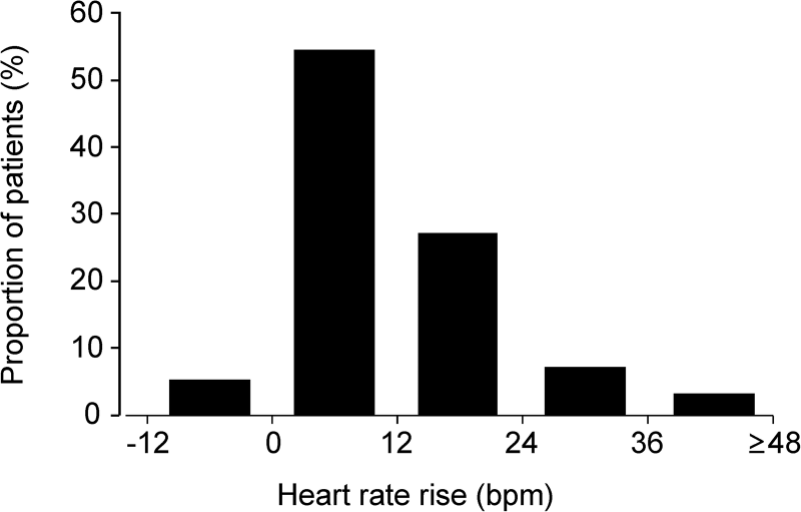
Distribution of changes in heart rate prior to loaded exercise.

Eight hundred and eighteen patients were recruited across both centres. Changes in heart rate while patients acclimatised to the exercise bike conditions at zero workload (unloaded cycling) were similar between centres (table 1; figure 2).

**Table 1:** Distribution of heart rate changes while patients acclimatised to the exercise bike conditions at zero workload (unloaded cycling)

Heart rate rise preloaded exercise (95% CI)	UCLH	Plymouth
Median	9 (9 to 10)	10 (9 to 11)
25th centile	5 (4 to 6)	5 (4 to 6)
75th centile	16 (15 to 17)	17 (15 to 19)

Data are shown as median (95% CIs).

Upper tertile values were similar to previous data showing that an abnormal EHRR ≥12 bpm was associated with an increased risk of sudden cardiac and all-cause cardiovascular death.^15^ We therefore explored this upper tertile cut-off value in subsequent analyses. In the UCLH cohort, 237/586 (40.4%) demonstrated EHRR ≥12 bpm (table 2). Cardiovascular drug therapy and co-morbidities were similar between patients with, or without, EHRR (see online supplementary tables S2 and S3). We did not find any relationship between EHRR≥12 bpm and the Revised Cardiac Risk Index (relative risk of RCRI≥2: 1.00 (95% CI 0.77 to 1.29); p=0.98). We observed similar results in a second (Plymouth) cohort, where 99/232 (42.7%) patients had EHRR (table 2).

**Table 2:** Demographics for both cohorts, stratified by heart rate change during unloaded cycling (zero workload) of patients acclimatised to the exercise bike conditions

	UCLH		Plymouth	
	Normal	EHRR	Normal	EHRR
Number (%)	349 (59.6)	237 (40.4)	133 (57.3)	99 (42.7)
Age (years)	61 (60 to 63)	65 (63 to 66)	65 (63 to 68)	67 (65 to 70)
Gender (male; %)	249 (71.3)	99 (41.8)	87 (65.4)	44 (45.8)
BMI (kg/m^2^)	27.0 (26.4 to 27.5)	28.5 (27.5 to 29.4)	27.5 (26.7 to 28.2)	29.1 (28.2 to 30.0)
Malignancy (n; %)	120 (45.6)	89 (50.9)	103 (77.4)	72 (75)

Data are shown as median (95% CIs).

Data analysed by one-way ANOVA or Fisher's exact test.

ANOVA, analysis of variance; BMI, body mass index; EHRR, exaggerated heart rate rise. University College London Hospitals NHS Trust.

We found that baseline and peak heart rates during exercise were not associated with EHRR (table 3). Consistent with this hyper-adrenergic pre-exercise phenotype, both systolic and diastolic blood pressure (measured before CPET) were higher in patients with EHRR (table 4). Although peak systolic blood pressure during exercise was similar between groups, the increase in systolic pressure from baseline was lower in patients with EHRR (table 4).

**Table 3:** CPET heart rate data, stratified by heart rate change during unloaded cycling (zero workload) of patients acclimatised to the exercise bike conditions

	Normal	EHRR	p Value
Pre-exercise			
Resting heart rate (per min)	82 (81 to 84)	82 (80 to 83)	0.75
Zero workload heart rate (per min)	88 (86 to 89)	104 (101 to 106)	<0.001
Heart rate change, zero workload (per min)	5 (5 to 6)	22 (20 to 23)	<0.001
Exercise			
Peak heart rate during CPET (per min)	134 (132 to 137)	135 (132 to 139)	0.56
Heart rate change, from baseline (per min)	51 (48 to 54)	53 (50 to 56)	0.60

Data are shown as mean (95% CIs).

Data analysed by one-way ANOVA.

ANOVA, analysis of variance; CPET, cardiopulmonary exercise testing; EHRR, exaggerated heart rate rise.

**Table 4:** Exercise-evoked changes in blood pressure

	Normal	EHRR	p Value
Baseline			
Systolic blood pressure (mm Hg)	144 (142 to 147)	157 (153 to 161)	<0.001
Diastolic blood pressure (mm Hg)	82 (81 to 84)	85 (83 to 87)	0.02
Exercise			
Peak systolic blood pressure (mm Hg)	189 (186 to 193)	192 (188 to 196)	0.22
Peak diastolic blood pressure (mm Hg)	89 (86 to 92)	95 (88 to 102)	0.11
Increase from baseline (systolic; mm Hg)	45 (42 to 48)	34 (31 to 37)	<0.001

Cohort from University College London Hospital: data are shown as mean (95% CIs).

Data analysed by one-way ANOVA.

ANOVA, analysis of variance; EHRR, exaggerated heart rate rise.

### Exercise-evoked ischaemia

In both centres, similar proportions of patients (27.3–40.4%) demonstrated ST-segment depression ≥1 mm during CPET. Continuous ECG recordings revealed an association between EHRR and ST-segment depression ≥1 mm in both cohorts (table 5). Across both centres, EHRR was associated with an increased relative risk of developing significant ST depression (relative risk: 1.7 (95% CI 1.3 to 2.1); p<0.001). Adjusting for changes in heart rate during exercise using the ST–heart rate (ST/HR) index, we again observed greater ST depression in EHRR patients (table 5). EHRR was not associated with exercise-evoked atrial and/or ventricular dysrhythmias (data not shown).

**Table 5:** ST-segment changes in both cohorts

	Normal	EHRR	p Value
ST change (mm)	−0.50 (−0.85 to −0.14)	−0.95 (−1.09 to −0.81)	<0.0001
ST/HR index (mm/min)	−0.01 (−0.02 to −0.01)	−0.02 (−0.03 to −0.02)	<0.0001

Data are shown as mean (95% CIs) for both cohorts.

Data analysed by one-way ANOVA.

ANOVA, analysis of variance; EHRR, exaggerated heart rate rise.

### Preoperative cardiopulmonary performance

Given we found an association between EHRR and ECG changes compatible with coronary artery dysfunction, we predicted that this occult sympathetic autonomic dysfunction phenotype should also be associated with impaired cardiopulmonary reserve. In addition to lower 
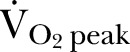
, we found that oxygen pulse—a robust surrogate for left ventricular function- was >10% lower in patients with EHRR (table 6). Left ventricular performance was more likely to fail to meet age-,weight and gender predicted norms in patients with EHRR (relative risk 1.26 (95% CI 1.14 to 1.39); p<0.001).

**Table 6:** Cardiopulmonary exercise testing data

	Normal	EHRR	p Value
Anaerobic threshold (mL/kg/min)	11.1 (10.8 to 11.4)	10.6 (10.2 to 11.1)	0.008
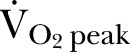 (% predicted)	78 (75 to 81)	74 (71 to 77)	0.05
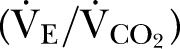	30.1 (29.6 to 30.7)	30.2 (29.5 to 30.9)	0.86
Oxygen pulse (% predicted)	95 (93 to 98)	85 (82 to 88)	0.0001

Data are shown as mean (95% CIs) for both cohorts.

Data analysed by one-way ANOVA.

ANOVA, analysis of variance; EHRR, exaggerated heart rate rise; 
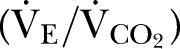
, ventilatory equivalents for carbon dioxide; 
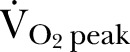
, peak oxygen consumption.

### Perioperative data

**Figure 3:**
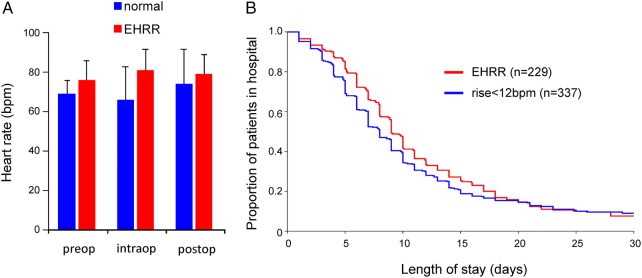
Perioperative associates of exaggerated heart rate responses pre-exercise. (A) Heart rate immediately preoperatively, at end of surgery and 30 min postextubation, stratified by exaggerated heart rate rise (EHRR). Two-way analysis of variance (operation time point×cardiopulmonary exercise testing heart rate phenotype) showed that EHRR (22/54 patients in POM-O trial) was independently associated with higher heart rates during the perioperative period (p=0.017). (B) Kaplan-Meier plot for length of hospital stay following major surgery, stratified by EHRR (n=566; p=0.03, by Gehan-Breslow-Wilcoxon survival analysis).

Patients with preoperative EHRR (n=22/54) had higher heart rates throughout the perioperative period (p=0.017; figure 3A). Outcomes data from the Plymouth and UCLH cohorts showed that EHRR was associated with longer hospital stay following major surgery (n=566; p=0.03, by Gehan-Breslow-Wilcoxon survival analysis; figure 3B).

## Discussion

These data demonstrate from two separate cohorts of surgical patients that the sympathetic autonomic response elicited during unloaded pedaling prior to ramped exercise (EHRR) is associated with increased risk of ECG-defined ischaemia, inferior cardiac performance and prolonged hospital stay. There is compelling physiological evidence to show that EHRR is chiefly due to mental stress. Similar rises in heart rate occur in patients sitting on a bike before exercise, but not pedalling, which are not accounted for by changes in posture.^15^ These data strongly suggest that EHRR is not due to pedalling-induced increased oxygen consumption, but rather to sympathetic activation due to stress. Our data add support to this assertion, since low aerobic capacity was evident regardless of presence/absence of EHRR. Many patients express anxiety at the time of CPET, presumably because of uncertainty about their ability to undergo an unfamiliar acute, vigorous physical challenge. This observation is further supported by higher resting blood pressure in patients with EHRR, even though a diagnosis of hypertension was similarly prevalent across the groups. Several studies using a different experimental paradigm have also identified that mental stress alone can trigger silent myocardial ischaemia.^10–14^ It is conceivable—and worthy of future investigation—that mental stressors such as task-oriented tests could similarly identify preoperative patients at the greatest risk of excessive sympathetic activity.

Previous studies have demonstrated that ST-segment depression to levels ≥1 mm independently predict future cardiac events in asymptomatic populations.^19^ We did not explore early heart rate changes during exercise, which have also been associated with excess cardiovascular risk. As a potent trigger of increased heart rate and acute endothelial dysfunction,^8^ exaggerated sympathetic outflow following minor, including mental, stress may be an important—though underappreciated—contribution to several morbidities observed commonly in the perioperative setting. Mental stress triggers myocardial ischaemia in patients with coronary artery disease, through pathological vasoconstriction following acetylcholine infusion.^10–14^ A minority of apparently otherwise healthy individuals who exhibit increases in heart rate as a result of the mental stress evoked by the thought of vigorous exercise are at increased risk of sudden cardiac death.^15^ In addition to well-documented consequences on myocardial ischaemia, it is increasingly recognised that excessive sympathetic activation can cause extracardiac cellular injury.^9^ High levels of endogenous catecholamines are likely to alter perioperative haemodynamic management, particularly in the absence of flow-guided monitoring.^34^ Hepatic dysfunction,^35^ acute lung injury^36^ and promotion of bacterial overgrowth^37^ provide direct and/or indirect mechanisms through which sympathetic activation can adversely influence postoperative outcomes. Persistently elevated plasma catecholamine levels also predispose to infection,^38^ through dysregulation of adhesion molecules,^39^ apoptosis^40^ and β-adrenoreceptor-mediated redistribution of lymphocytes from peripheral blood to lymphatic tissue.^41^ Consistent with these translational insights, intraoperative tachycardia and hypertension have been associated with postoperative morbidity and prolonged hospital stay after major non-cardiac surgery.^42^

Strengths of these data are that all analyses were performed blinded to primary and secondary outcomes, in two separate centres. Describing this dysautonomic parameter in the context of a highly phenotyped cardiovascular test enables correlation with comprehensive cardiopulmonary physiological data. Established biological plausibility for this phenomenon and preceding similar findings, albeit with different testing methodology in non-operative patients, lends important support. Interindividual genetic variation in adrenergic receptor and signalling may influence these responses.^43^ The observational nature and lack of an intervention limit more robust conclusions.

There are several clinical implications raised by these data. A clearer understanding is required of which patients may benefit from perioperative sympatholysis. Identifying highly phenotyped patients at the highest perioperative risk of excess sympathetic activation could provide a new rationale for targeted sympatholysis, rather than a ‘one-size-fits-all’ approach which does not appear to have an acceptable therapeutic risk-benefit ratio.^2^ ^3^ Our data are consistent with recent experimental rodent data identifying off-target risks of β-blockade following perioperative anaemia.^44^ The subpopulation of patients we have identified who exhibit high sympathetic activity to stress, frequently driven by perioperative factors including acute blood loss and/or hypovolaemia, may have an increased risk for vital organ hypoxia and injury if sympatholysis was implemented given their established cardiac dysfunction. Certainly, sympatholysis in these patients could play an important role in perioperative stroke, particularly given the importance of avoiding hypotension.^45^ Taken together, these studies suggest that in addition to the need for further trials,^46^ a mechanistic re-evaluation of the appropriate clinical indications and timing for perioperative sympatholysis is necessary to mitigate the detrimental effects of β-blockade and α-2 agonism identified by serial POISE trials.

In summary, we have identified a significant number of patients who exhibit cardiovascular changes associated with excess sympathoadrenal activity in the preoperative setting. These patients develop exercise-induced, ECG-defined ischaemia and sustain prolonged hospital stay. This subset of patients may benefit from interventions designed to counteract the multiorgan, deleterious impact of excessive sympathetic activity and/or inappropriately targeted sympatholysis on cellular function.
